# Exosomes Derived From M2b Macrophages Attenuate DSS-Induced Colitis

**DOI:** 10.3389/fimmu.2019.02346

**Published:** 2019-10-16

**Authors:** Ruibing Yang, Yao Liao, Lifu Wang, Ping He, Yuanjia Hu, Dongya Yuan, Zhongdao Wu, Xi Sun

**Affiliations:** ^1^Medical Department of Xizang Minzu University, Xianyang, China; ^2^Department of Parasitology of Zhongshan School of Medicine, Sun Yat-sen University, Guangzhou, China; ^3^Key Laboratory of Tropical Disease Control, Ministry of Education, Sun Yat-sen University, Guangzhou, China; ^4^Provincial Engineering Technology Research Center for Biological Vector Control, Guangzhou, China; ^5^Key Laboratory for Basic Research in Life Sciences, Institutions of Higher Learning, Xianyang, China; ^6^State Key Laboratory of Quality Research in Chinese Medicine, Institute of Chinese Medical Sciences, University of Macau, Macau, China

**Keywords:** M2b macrophage, IL-1β, exosomes, inflammatory bowel disease, CCL1/CCR8 axis

## Abstract

Macrophages are commonly classified as M1 macrophages or M2 macrophages. The M2 macrophages are further sub-categorized into M2a, M2b, M2c, and M2d subtypes. The M2a, M2b, and M2c subtypes play roles in anti-inflammatory activity, tissue remodeling, type 2 T helper cell (Th2) activation, and immunoregulation. Previous studies have shown that macrophage exosomes can affect some disease processes. Exosomes are 30–150-nm lipid bilayer membrane vesicles derived from most living cells, with important biological functions. The role of exosomes in preventing the development of autoimmune diseases, including inflammatory bowel disease (IBD), has evoked increasing interest. Here, we analyze the roles of exosomes derived from M2a, M2b, and M2c macrophage phenotypes in dextran sulfate sodium (DSS)-induced colitis. Exosomes were isolated from the supernatant of different types of macrophages and identified via transmission electron microscopy (TEM), western blotting, and NanoSight. The results showed that M2b macrophage exosomes significantly attenuated the severity of DSS-induced colitis in mice. The number of regulatory T (Treg) cells in the spleens of mice with colitis and levels of IL-4 both increased following treatment with M2b macrophage exosomes. In addition, key cytokines associated with colitis (IL-1β, IL-6, and IL-17A) were significantly suppressed, following treatment with M2b macrophage exosomes. The M2b macrophage exosomes exerted protective effects on DSS-induced colitis, mainly mediated by the CC chemokine 1 (CCL1)/CCR8 axis. These findings provide a novel approach for the treatment of IBD.

## Introduction

Inflammatory bowel disease (IBD, including Crohn's disease and ulcerative colitis) is considered to be the result of chronic and remittent-relapsing intestinal inflammation and intestinal tract destruction (1). The condition represents a major global health care burden that affects millions of people worldwide, especially in Europe and North America, where its prevalence is the highest, and in Asia, where its incidence has been rising in recent years (2–4). The exact etiologies of IBD remain unknown. However, over the past few decades, the main factors responsible for Crohn's disease and ulcerative colitis are generally believed to include environmental changes, genetic make-up, abnormalities in the gut microbiota, and dysregulated immune responses (5, 6). Chronic inflammation is a dysregulated immune response; therefore, many investigations into the pathogenesis of IBD have been focused on immune abnormalities (5). Rectal bleeding, abdominal discomfort, diarrhea, and weight loss are the most common symptoms of IBD (7). Traditional treatments for IBD primarily consist of 5-aminosalicylic acid agents, steroids, and antimicrobials. However, these drugs have limitations and can induce adverse events. Within the past two decades, biological therapies have revolutionized the treatment of IBD, while demonstrating a higher efficacy and safety profile (8–10).

Macrophages are heterogeneous, circulate in the blood, or are concatenated in different organs and tissues, and constitute the first barrier against disease (11). The phenotype and functions of macrophages are regulated by the surrounding microenvironment (12). Macrophages are commonly classified into two distinct subsets based on the phenotype: (1) classically activated (M1) macrophages, which are typically induced by bacterial lipopolysaccharide (LPS) or type 1 T helper cell (Th1) cytokines, such as interferon (IFN)-γ and tumor necrosis factor (TNF)-α. The M1 macrophages secrete higher levels of pro-inflammatory cytokines, such as TNF-α, interleukin (IL)-1α, IL-12, IL-23, IL-1β, and IL-6. (2) Alternatively, activated (M2) macrophages are anti-inflammatory and polarized by type 2 T helper cell (Th2) cytokines (IL-4, IL-13, and IL-33). The M2 macrophages produce higher levels of the immunoregulatory cytokine, IL-10 (12, 13). Furthermore, depending on the activating stimulus, M2 macrophages can be classified as M2a, M2b, M2c, or M2d subtypes (14, 15). The M2a macrophages can be induced by IL-4 and IL-13 and play a role in anti-inflammatory activity and tissue remodeling (12, 14, 15). The M2b macrophages can be induced by stimulation with immune complexes, toll-like receptor (TLR) agonists, or IL-1 receptor ligands, and play a role in Th2 activation and immunoregulation (12, 14). The M2c subset of macrophages is induced by glucocorticoids, IL-10, or transforming growth factor (TGF)-β, and exhibits anti-inflammatory activities by further releasing IL-10 and TGF-β (16, 17). The M2d macrophage is induced by TLR agonists through the adenosine receptor and plays a role in angiogenesis and tumor progression (14).

Exosomes are 30–150-nm lipid bilayer membrane vesicles, which are derived from most living cells and released into the extracellular medium (18). They carry proteins, genes (mRNA, miRNA, and DNA), and lipids *in vitro* and *in vivo*, and have important biological functions (18). In recent years, the function and potential application of exosomes in IBD has evoked increasing interest. Previous studies have demonstrated that exosomes derived from dendritic cells (19), human umbilical cord mesenchymal stem cells (20), and granulocytic myeloid-derived suppressor cells (21) can attenuate colitis. Because exosomes have less biohazardous potential and cytotoxicity, and are not easily degraded, they have more advantages over parental cells (22). Furthermore, because M2 macrophages are associated with anti-inflammatory and immunoregulatory activity, we hypothesized that the combined advantages of exosomes derived from various M2 macrophage phenotypes could be useful in the treatment of IBD. Moreover, the specific subtype of M2 macrophages with the best effects remains unknown.

In this study, M2 macrophage exosomes (M2a, M2b, and M2c) were isolated from the supernatant of different types of macrophages and the effects of these exosomes in IBD were evaluated. We found that M2 macrophage exosomes attenuated the severity of dextran sulfate sodium (DSS)-induced colitis, and exosomes derived from the M2b macrophages were more effective than those derived from M2a and M2c macrophages.

## Materials and Methods

### Animals and Ethics

Male BALB/c mice, aged 6 weeks (weighing 18–20 g), were purchased from the Experimental Animal Center of Guangdong. All animal experimental procedures were approved by the Animal Research Ethics Committee of Sun Yat-sen University and conformed to the Chinese National Institute of Health Guide for the Care and Use of Laboratory Animals.

### Macrophage Generation and Exosome Purification

The femur and tibia of BALB/c mice were removed, left in 75% ethanol for 5 min, and then washed in Dulbecco's modified Eagle's medium (DMEM). Cells within the marrow were prepared in a single cell suspension and cultured in DMEM (GIBCO, Germany) with 10% (v/v) fetal bovine serum, 100 U/mL of penicillin (Sigma, Germany), 100 μg/mL of streptomycin (Sigma, Germany), 1 mM L-glutamine (Sigma, Germany), and recombinant murine macrophage colony-stimulating factor (M-CSF) (20 ng/mL, PeproTech, USA). On the third and fifth days, half of the cell culture supernatant was discarded, and an equivalent volume of the medium was added. On the seventh day, bone marrow-derived macrophages (BMDMs) were harvested. The BMDMs were either stimulated with IL-13 (R&D Systems, USA), IL-10 (R&D Systems), or IL-1β (R&D Systems) or left untreated. The resulting culture supernatant and cells were harvested 24 h post-stimulation. Exosomes from the supernatant of the cell culture media were purified using an exosome isolation kit (Invitrogen, USA), according to the manufacturer's instructions.

### Electron Microscopy, NanoSight, and Western Blotting

Exosomes were analyzed using negative-staining transmission electron microscopy (TEM). The exosomes were suspended in 2% glutaraldehyde and loaded onto copper grids, after which they were negatively stained with 3% (w/v) aqueous phosphotungstic acid for 1 min. The grids were examined using the FEI Tecnai G2 Spirit Twin transmission electron microscope. In addition, macrophage exosomes were analyzed using the NanoSight NS300 instrument (Malvern Instruments, UK).

The markers CD63, CD9, CD81, CCL1 of exosomes and CCL1, CCR8, and IL-4 of the mice colon were detected by western blotting. The standard sodium dodecyl sulfate-polyacrylamide gel electrophoresis (SDS-PAGE) procedure was followed, macrophage exosomes were lysed in a western blotting lysis buffer and separated using 10% SDS-PAGE. Subsequently, proteins were electrophoretically transferred onto a polyvinylidene fluoride blotting membrane (GE Healthcare Life Sciences, UK), and then blocked using 5% skimmed milk. The membranes were incubated with anti-mouse CD63, CD9, CD81, CCL1, CCR8, and IL-4 antibody (BD Biosciences, USA) overnight at 4°C. Anti-mouse antibodies conjugated with horseradish peroxidase (HRP) (Jackson Immunoresearch Labs Inc., USA) were used as secondary antibodies. Membranes were visualized via an enhanced chemiluminescence (ECL) chemiluminescent detection system (Amersham, USA).

### Induction of Colitis and Treatment

Acute colitis was induced by administering water with 3% (wt/vol) DSS (molecular mass 36–50 kDa; MP Biomedicals, Illkirch, France) to mice over 8 days (days 1–8). The control mice received regular drinking water. The cells of BMDMs were either unstimulated (M0) or stimulated with IL-13 (M2a), IL-10 (M2c), or IL-1β (M2b), and subsequently administered via intraperitoneal (i.p.) injection (1 × 10^6^) on day 1. Exosomes purified from different macrophage phenotypes (M0, M2a, M2b, and M2c) were administered via i.p. injection (50 mg per mouse) from days 1 to 8. The same volume of the vehicle (phosphate-buffered saline, PBS) was administered to the control group via i.p. injection.

### Clinical Scoring of Disease

Mice were observed daily during treatment. Any changes in body weight, or occurrence of diarrhea or bleeding were recorded. Bleeding was determined using the Hemoccult assay kit (Nanjing Jiancheng Bio-engineering Institute, China). The clinical disease score (disease activity index, DAI) was evaluated based on weight loss, diarrhea, and bleeding, as described by Sann et al. (Table 1) (23).

**Table 1:** Scores of the disease activity index (DAI).

**Score**	**Body weight loss (%)**	**Bleeding**	**Diarrhea**
0	<2%	No blood in stool	Normal stool
1	≥2–<5%	Weak hemoccult in stool	Softer stool
2	≥5–<10%	Visible blood in stool	Unformed stool
3	≥10–<15%	Fresh rectal bleeding	Watery stool
4	≥15%	–	–

### Macroscopic Assessment and Histological Evaluation

Mice were sacrificed on day 8 and their colons were resected. Colon length (an indirect marker of inflammation) was measured, and the macroscopic characteristics of colons were assessed by an independent observer blinded to the study. The macroscopic scores were assessed using the following parameters: hyperemia, wall thickening, ulceration, and extent of inflammation and damage (Table 2) (24).

**Table 2:** Assessment of macroscopic scores.

**Score**	**The damage of colon**
0	No damage
1	Hyperemia without ulcers
2	Hyperemia and wall thickening without ulcers
3	One ulceration site without wall thickening
4	Two or more ulceration sites
5	0.5 cm extent of inflammation or major damage
6–10	1 cm extent of inflammation or severe damage

Colons were fixed overnight with 4% (w/v) paraformaldehyde and then embedded in paraffin. Five-micrometer paraffin-embedded colon sections were prepared and stained with hematoxylin and eosin (H & E). Histopathological scores were determined in a blinded fashion, according to the criteria described by Sann et al. (Table 3) (23). The histopathological scores of colonic lesions were assessed based on the extent of inflammation, neutrophilic and lympho-histiocytic infiltration, crypt damage, crypt abscess formation, sub-mucosal edema, loss of goblet cells, and reactive epithelial hyperplasia. The histopathological scores were determined by adding all scores of the aforementioned parameters.

**Table 3:** Histopathological scores.

**Score**	**Inflammation**	**Infiltration neutrophils + lympho-histiocytes**	**Crypt damage**	**Crypt abscess**	**Sub-mucosal edema**	**Loss of goblet cells**	**Reactive epithelial hyperplasia**
0	None	None	None	None	None	None	None
1	Mucosa	Focal	Basal 1/3	Focal	Focal	Focal	Focal
2	Mucosa + submucosa	Multifocal	Basal 2/3	Multifocal	Multifocal	Multifocal	Multifocal
3	Mucosa + submucosa + muscle layer	Diffuse	Entire crypt damage	–	Diffuse	Diffuse	Diffuse
4	Transmural	–	Crypt damage + ulceration	–	–	–	–

### Flow Cytometry

To determine the splenic regulatory T (Treg) subset levels, cells were isolated from the spleens of mice, washed in PBS, and stained with fluorochrome-conjugated antibodies against the cell surfaces of CD3e, CD4, and CD25 (BD Biosciences, USA). The antibodies were used at concentrations recommended by the manufacturers. Cells were then fixed and permeabilized using a fixation/permeabilization solution, according to the manufacturer's instructions, and stained with Foxp3. Images of all samples were acquired on a CytoFLEX S flow cytometer (Beckman Coulter, USA). The numbers of Tregs in the spleens were quantified and expressed as percentages of the CD3e^+^CD4^+^CD25^+^Foxp3^+^ cell population.

### RNA Extraction and Real-Time Polymerase Chain Reaction (PCR)

Total RNA was isolated using the Trizol reagent (Invitrogen, USA). Furthermore, RNA was quantified by measuring the ratio of the absorbance at 260 and 280 nm using the NanoDrop ND-2000 spectrophotometer (Thermo Scientific, USA). The RNA was converted to complementary DNA (cDNA) using a Thermo Scientific Revert Aid First Strand cDNA Synthesis Kit (Thermo Scientific, USA), according to the manufacturer's instructions. Furthermore, reverse transcription PCR was performed using SYBR Green QPCR Master Mix (TaKaRa, Japan). The reaction comprised an initial denaturation at 95°C for 30 s, followed by amplification for 35 cycles at 95°C for 5 s, and 60°C for 20 s. The primers of IL-1β, IL-6, IL-17A, CCL1, CCR8, IL-4, and glyceraldehyde-3-phosphate dehydrogenase (GAPDH) are listed in Table 4.

**Table 4:** Primers used for real-time PCR analysis.

**Genes**	**Primer**	**Sequence (5^′^→3^′^)**
IL-1β	Forward primer	CTCACAAGCAGAGCACAAGC
	Reverse primer	TCCAGCCCATACTTTAGGAAGA
IL-6	Forward primer	TAGTCCTTCCTACCCCAATTTCC
	Reverse primer	TTGGTCCTTAGCCACTCCTTC
IL-17A	Forward primer	GCTCCAGAAGGCCCTCAGACT
	Reverse primer	CCAGCTTTCCCTCCGCATTGA
CCL1	Forward primer	GGATGTTGACAGCAAGAGCA
	Reverse primer	ACAGGAGGAGCCCATCTTTC
CCR8	Forward primer	TGGTGCTCACCGTAGTCATT
	Reverse primer	TCCATCCAAGATGTGCAGGT
IL-4	Forward primer	AACGAGGTCACAGGAGAAGG
	Reverse primer	CTGCAGCTCCATGAGAACAC
GAPDH	Forward primer	ACTCCACTCACGGCAAATTC
	Reverse primer	TCTCCATGGTGGTGAAGACA

### *Ex vivo* Fluorescence Imaging of Exosomes in the Colon

Exosomes were stained with DiR (1,1′-dioctadecyl-3,3,3′,3′-tetramethylindotricarbocyanine iodide), and then centrifuged at 120,000 *g* for 1.5 h at 4°C in an Optima L-100xp tabletop ultracentrifuge (Beckman, USA) to wash the unstained DiR. Mice were injected with DiR labeled exosomes. Mice were then sacrificed 12 h later, their colons were resected, and fluorescence images were obtained using an *in vivo* imaging system (Maestro, USA).

### Immunohistochemistry

Immunohistochemistry was performed to evaluate CCL1, CCR8, and IL-4 protein expression levels in the colon. The paraffin sections were deparaffinized and antigen retrieval was performed by irradiating the samples in a microwave. The sections were incubated with 3% H_2_O_2_ to block endogenous peroxidase activity, after which they were blocked with 1% bovine serum albumin and incubated with anti-mouse CCL1, CCR8, and IL-4 antibodies (1:100) overnight at 4°C. The sections were then incubated with the secondary antibody. HRP activity was detected by 3, 3′diaminobenzidine (DAB). The sum of the integrated optical density (IOD) was analyzed using the Image-Pro Plus 6.0 software.

### Statistical Analyses

Data were expressed as mean ± standard error of the mean (SEM). One-way analysis of variance (ANOVA), followed by the Dunnett's *post hoc* test were used to analyze the various groups. *P* < 0.05 was considered statistically significant.

## Results

### Isolation and Identification of Macrophage Exosomes

**Figure 1:**
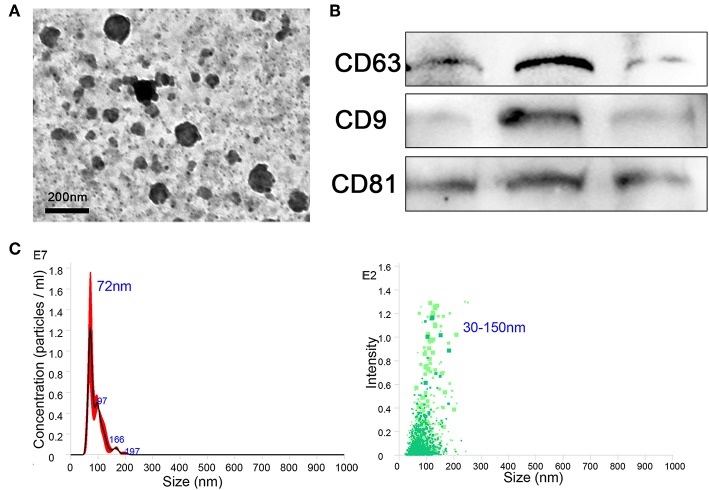
Exosomes were purified from the supernatant of macrophages. **(A)** Exosomes were analyzed by negative-staining transmission electron microscopy (TEM). **(B)** Exosome specific markers CD63, CD9, and CD81 were detected by western blotting. **(C)** The size distribution profile of the exosomes was analyzed by NanoSight.

The BMDMs were cultured *in vitro* and treated with IL-13, IL-10, and IL-1β to induce the phenotypes of M2a, M2c, and M2b, respectively, and M0 (untreated) was considered the control. After 24 h, culture supernatants were collected, and exosomes were extracted from the supernatants using extraction kits (from cell culture media), according to the manufacturer's instructions. The purity, quality, and morphology of the exosomes were analyzed using negative-staining TEM. Isolated macrophage exosomes were observed to have closed round vesicles with a typical diameter of 30–150 nm (Figure 1A). Furthermore, the expression of exosome markers CD63, CD9, and CD81 was detected by western blotting (Figure 1B). In addition, NanoSight was used to investigate the distribution profile of macrophage exosomes and revealed a peak of 72 nm (Figure 1C). These data indicate that the exosomes were successfully isolated from the culture supernatants.

### M2 Macrophage Exosomes Attenuate the Clinical Scores of Mice With DSS-Induced Colitis

To assess the effect of M2 macrophage exosomes on the development of colitis, the DSS-induced colitis model was established. The following groups of mice were used: water + PBS; DSS + PBS; DSS + M0-cell; DSS + IL-13-cell; DSS + IL-10-cell; DSS + IL-1β-cell; DSS + M0-exo; DSS + IL-13-exo; DSS + IL-10-exo; and DSS + IL-1β-exo. The DSS + M0-cell; DSS + IL-13-cell; DSS + IL-10-cell; and DSS + IL-1β-cell groups were injected (i.p.) with cells (1 × 10^6^) of BMDMs that were either unstimulated (M0), or stimulated with IL-13 (M2a), IL-10 (M2c), or IL-1β (M2b) on day 1. The DSS + M0-exo; DSS + IL-13-exo; DSS + IL-10-exo; and DSS + IL-1β-exo groups were injected (i.p.) with exosomes purified from macrophage phenotypes of M0 (unstimulated); M2a (stimulated with IL-13); M2b (stimulated with IL-1β); and M2c (stimulated with IL-10) from days 1 to 8. The water + PBS and DSS + PBS groups were injected (i.p.) with a similar volume of vehicle (PBS).

**Figure 2:**
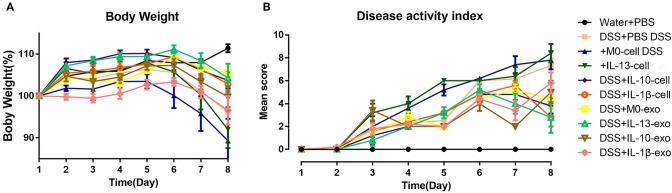
Body weight and disease activity index (DAI) of mice with acute dextran sulfate sodium (DSS)-induced colitis treated with macrophage exosomes. The following groups of mice were used in the study: water + phosphate-buffered saline (PBS); DSS + PBS; DSS + M0-cell; DSS + IL-13-cell; DSS + IL-10-cell; DSS + IL-1β-cell; DSS + M0-exo; DSS + IL-13-exo; DSS + IL-10-exo; and DSS + IL-1β-exo. **(A)** Daily changes in body weight of each group. **(B)** Changes in DAI scored from body weight loss, diarrhea, and bleeding.

The clinical disease score was evaluated based on weight loss, diarrhea, and bleeding. As shown in Figure 2A, mice of the DSS + PBS group lost weight over time, and weight loss in mice with colitis that were treated with M2 macrophages (DSS + IL-13-cell; DSS + IL-10-cell; and DSS + IL-1β-cell groups) was alleviated. In addition, weight loss in mice with colitis that were treated with M2 macrophage exosomes (DSS + M0-exo; DSS + IL-13-exo; DSS + IL-10-exo; and DSS + IL-1β-exo) was also alleviated. The DSS treatment caused severe diarrhea and bleeding in mice with colitis. Moreover, M2b and M2c macrophages, and M2a, M2b, and M2c macrophage exosomes alleviated the severity of diarrhea and bleeding. The effects of M2 macrophage exosomes were superior to those of M2 macrophages. The DAI of mice with colitis that were treated with M2 macrophage exosomes was lower than the DAI of those treated with PBS, M0 macrophages, or M2 macrophages (Figure 2B).

### M2b Macrophage Exosomes Ameliorate Reduction of the Colon Length and Macroscopic Scores in DSS-Induced Colitis

**Figure 3:**
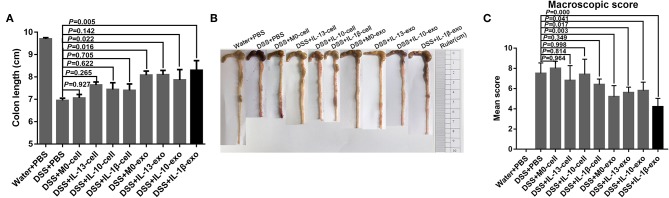
Colon length and colon macroscopic scores of mice treated with macrophage exosomes. **(A)** Mice were sacrificed on day 8 and colon lengths (an indirect marker of inflammation) were measured. **(B)** Macroscopic appearance of colons. **(C)** Mean macroscopic scores of colons. Macroscopic scores were assessed by hyperemia, wall thickening, ulceration, and extent of inflammation and damage.

Colon length can be significantly reduced by DSS in the mouse model. To evaluate the therapeutic potential of M2 macrophage exosomes in mice with DSS-induced colitis, the colon length was evaluated. The results showed that the colons of mice with colitis, which were treated with M2b macrophage exosomes were longer than those of other groups of mice with colitis (Figures 3A,B, Table 5). The macroscopic scores were assessed by hyperemia, wall thickening, ulceration, and the extent of inflammation and damage. The mean macroscopic scores of mice with colitis that were treated with M2b macrophage exosomes were lower than those of other groups of mice with colitis (Figure 3C, Table 5). These results demonstrate that M2b macrophage exosomes more effectively ameliorate the reduction of colon length and reduce macroscopic scores in mice with DSS-induced colitis than the exosomes of other macrophage phenotypes and macrophage cells.

**Table 5:** Values of the evaluation indexes.

**Group**	**DAI on day 8 (mean)**	**Colon length (cm) (mean)**	**Macroscopic scores (mean)**	**Histopathological score (mean)**	**Treg subset levels (mean)**	***n***
Water + PBS	0.0	9.7	0.0	0.0	10.3	6
DSS + PBS	7.3	6.9	7.5	12.5	9.3	6
DSS + M0-cell	7.8	7.1	8.0	12.0	9.6	6
DSS + IL-13-cell	2.8	7.6	6.8	14.4	10.1	6
DSS + IL-10-cell	8.4	7.4	7.4	10.2	10.0	6
DSS + IL-1β-cell	3.8	7.4	6.4	5.8	8.3	6
DSS + M0-exo	4.4	8.1	5.2	14.4	8.8	6
DSS + IL-13-exo	5.0	8.1	5.6	11.0	10.7	6
DSS + IL-10-exo	2.8	7.9	5.8	10.4	13.5	6
DSS + IL-1β-exo	5.8	8.3	4.2	4.0	12.7	6

### M2b Macrophage Exosomes Alleviate Colon Damage in Mice With DSS-Induced Colitis

**Figure 4:**
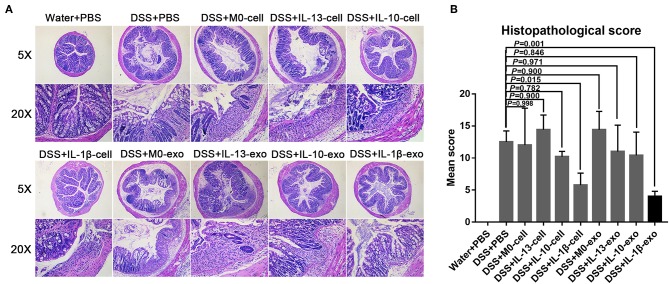
Histopathological changes in the colons of mice treated with macrophage exosomes. **(A)** Colon sections were examined with hematoxylin and eosin (H & E) (5× and 20×). **(B)** Histopathological scores were determined in a blinded fashion.

The results of histological assessment showed that DSS induced a significant inflammatory response, including inflammatory cell infiltration, crypt loss, sub-mucosal edema, crypt abscess formation, goblet cell loss, and reactive epithelial hyperplasia (Figure 4A). The results of H&E histopathology indicated that mice with colitis that were treated with M2b macrophage exosomes had significantly reduced inflammatory responses, compared with those treated with the exosomes of other macrophage phenotypes and macrophage cells (Figure 4A). Consistent with the histological assessment, histopathological scores, determined in a blinded fashion, in the colons of the groups treated with M2b macrophage exosomes were significantly lower than those in the colons of groups treated with the exosomes of other macrophage phenotypes and macrophage cells (Figure 4B, Table 5). Taken together, these data indicate that M2b macrophage exosomes could alleviate colon damage in mice with DSS-induced colitis, and their effects are superior to those of the exosomes of other macrophage phenotypes and macrophage cells.

### M2b Macrophage Exosomes Increased Treg Percentages in Mice With DSS-Induced Colitis

**Figure 5:**
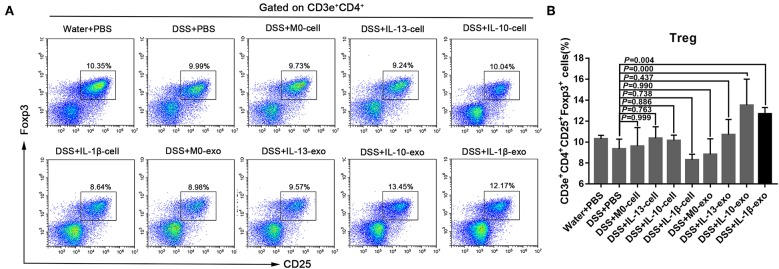
Splenic Treg subset levels in mice with experimental colitis. **(A)** Cells were isolated from the spleens of mice and stained with fluorochrome-conjugated antibodies against CD3e, CD4, CD25, and Foxp3. The number of Tregs in the spleens were expressed as percentages of the CD3e^+^CD4^+^CD25^+^Foxp3^+^ cell population. **(B)** Statistical analysis of Tregs.

The Treg subsets play an important role in regulating immune homeostasis and tolerance. In IBD, Treg subsets are linked to the development of inflammatory disorders. To determine the Treg subset levels, cells were isolated from the spleens of mice, and stained with fluorochrome-conjugated antibodies against CD3e, CD4, CD25, and Foxp3. The changes in Treg subset levels were evaluated by flow cytometry. We noted that the percentages of CD3e^+^CD4^+^CD25^+^Foxp3^+^ cells in the spleens of mice with DSS-induced colitis that were treated with M2b macrophage exosomes were significantly increased (12.7 ± 0.7%; *P* < 0.05), compared with those in the spleens of PBS-treated mice with DSS-induced colitis (9.3 ± 1%) (Figure 5, Table 5). In addition, the percentages of CD3e^+^CD4^+^CD25^+^Foxp3^+^ cells in the spleens of mice with DSS-induced colitis that were treated with M2c macrophage exosomes were significantly increased (13.5 ± 3%; *P* < 0.001) compared with PBS-treated mice with DSS-induced colitis (9.3 ± 1%) (Figure 5, Table 5). These findings indicate that M2b and M2c macrophage exosomes had protective effects against DSS-induced colitis mediated by an increased number of Treg cells.

### M2b Macrophage Exosomes Regulate Inflammatory Cytokine Production in Mice With DSS-Induced Colitis

**Figure 6:**
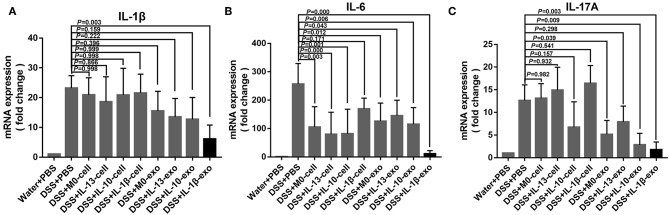
Inflammatory cytokine expression in the colons of mice was determined by real-time PCR; the housekeeping gene, *GAPDH*, was used as an internal reference. **(A)** The *IL-1*β mRNA expression levels. **(B)** The *IL-6* mRNA expression levels. **(C)** The *IL-17A* mRNA expression levels.

In IBD, levels of the pro-inflammatory cytokines, IL-1β, IL-6, and IL-17A are predictably increased in the mucosa. To determine whether macrophage exosomes can regulate IL-1β, IL-6, and IL-17A production in mice with DSS-induced colitis, real-time PCR was used to examine the expression of IL-1β, IL-6, and IL-17A. In the DSS + PBS group, IL-1β, IL-6, and IL-17A showed high levels of expression (Figure 6). Compared with the DSS + PBS group, IL-1β, IL-6, and IL-17A expression levels were reduced, following treatment with M2b macrophage exosomes (Figure 6). In addition, the expression levels of IL-1β, IL-6, and IL-17A in the groups treated with M2b macrophage exosomes were lower than those in groups treated with the exosomes of other macrophage phenotypes and macrophage cells (Figure 6). These results indicate that M2b macrophage exosomes can regulate inflammatory cytokine production in mice with DSS-induced colitis.

### M2b Macrophage Exosomes Carrying CCL1 Interact With CCR8 to Regulate Immunity in Mice With DSS-Induced Colitis

**Figure 7:**
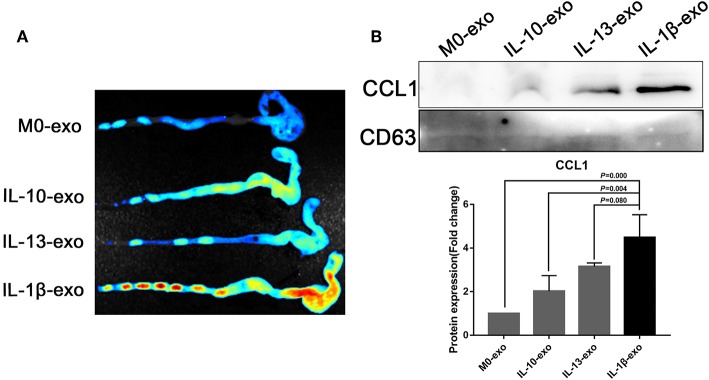
*Ex vivo* fluorescence imaging of exosomes in the colon; the CCL1 protein in exosomes was detected by western blotting. **(A)** *Ex vivo* fluorescence imaging of the distribution of DiR labeled exosomes in the colon. **(B)** Western blotting analysis of CCL1 protein expression in exosomes purified from various macrophage phenotypes, exosome marker CD63 used as internal reference.

**Figure 8:**
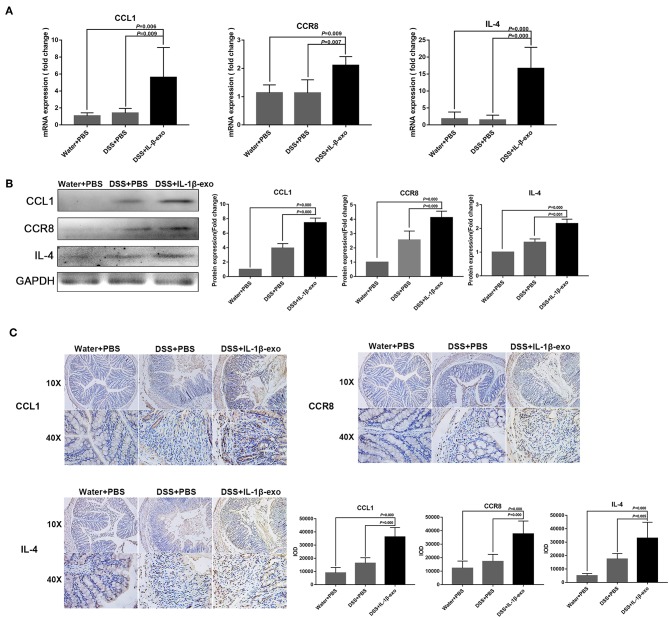
CCL1, CCR8, and IL4 expression in the colons of mice treated with M2b macrophage exosomes. **(A)** The CCL1, CCR8, and IL4 mRNA expression levels in the colons of mice were determined by real-time PCR; the housekeeping gene, *GAPDH*, was used as an internal reference. **(B)** Western blotting analysis of CCL1, CCR8, and IL4 protein expression in the colons of mice. **(C)** CCL1, CCR8, and IL4 expression in colon tissue was detected by immunohistochemistry, and the sum of the integrated optical density (IOD) was analyzed.

The M2b macrophages are characterized specifically by the secretion of the CCL1 chemokine. Furthermore, CCL1 can interact with CCR8, which is expressed on Th2 cells and Treg cells for immune regulation. Therefore, we questioned whether M2b macrophage exosomes carrying the CCL1 protein to the colon, interact with the CCR8 receptor to regulate Th2 polarization and Treg cells, and thereby attenuate DSS-induced colitis. As expected, *ex vivo* fluorescence imaging showed that macrophage exosomes arrived the colon of mice (Figure 7A). In addition, expression of the CCL1 protein in the exosomes was analyzed by western blotting. The CCL1 protein expression in M2b macrophage exosomes was significantly higher than that in other exosomes (Figure 7B). Real-time PCR demonstrated higher expression of CCL1 and CCR8 in the colon, following treatment with M2b macrophage exosomes (Figure 8A). The expression of IL-4, which was mainly produced by Th2 cells, was also increased following treatment with M2b macrophage exosomes (Figure 8A). The CCL1, CCR8, and IL-4 protein expression levels were confirmed by western blotting, and the results were consistent with those of real-time PCR (Figure 8B). Furthermore, the protein expression levels of CCL1, CCR8, and IL-4 were detected by immunohistochemistry, and found to be significantly increased following treatment with M2b macrophage exosomes, compared with the PBS treatment (Figure 8C).

Taken together, these data indicate that M2b macrophage exosomes carrying the CCL1 protein to the colon interact with CCR8 to promote IL-4 expression (Th2 polarization) and increase Treg percentages (Figure 5). The Th2-type immune response balanced the Th1-type immune response induced by DSS administration, and Treg cells balanced the inflammatory disorders of DSS-induced colitis, thereby relieving the colitis.

## Discussion

In this study, we assessed whether exosomes derived from different types of M2 macrophage phenotypes could have protective effects against the development of DSS-induced colitis. As expected, the current data demonstrate that body weight loss and the DAI are both reduced after treatment with M2a, M2b, and M2c macrophage exosomes. Moreover, after treatment with M2 macrophage exosomes, the colon length was improved in mice with colitis, and the mean macroscopic scores in the colon were decreased. Furthermore, M2b macrophage exosomes were more effective than M2a and M2c macrophage exosomes. Histological examination and scores showed that M2b macrophage exosomes alleviated colon damage in mice with DSS-induced colitis. In addition, M2b macrophage exosomes increased the percentage of Treg cells in mice with DSS-induced colitis and reduced pro-inflammatory cytokine (IL-1β, IL-6, and IL-17A) production in the colon of mice with DSS-induced colitis. Additionally, we found that M2b macrophage exosomes, carrying the CCL1 protein to the colon, interact with CCR8 to promote Th2 polarization and increase Treg percentages, thereby relieving colitis.

Innate immune responses are mediated by a variety of cell types, including neutrophils, monocytes, macrophages, dendritic cells, and epithelial, endothelial, and mesenchymal cells as the first line of defense (25). The infiltration of the gut mucosa and epithelium by polymorphonuclear leukocytes constitute the earliest signs of intestinal inflammation, and this infiltration persists throughout the course of IBD (26). In addition to innate immunity, another important factor affecting IBD is an imbalance in adaptive immunity. Effector T cells, including Th1, Th2, and Th17, have plasticity and can quickly adapt to stimulation by the surrounding environment, which is closely associated with the pathogenesis of IBD (27). The Treg cells are essential for the development of tolerance to self- and non-self-antigens (28). Given the importance of Treg cells in innate and adaptive immunity, defects in their functions underlie autoimmune, infectious, and chronic inflammatory conditions, including IBD (29). Moreover, imbalances in natural killer (NK) cells, natural killer T (NKT) cells, and innate lymphocytes (ILC) in the intestinal mucosa can also be important factors in the pathogenesis of IBD. Therefore, anti-inflammatory and immunoregulatory activities are key targets for the treatment of IBD.

Exosomes transport proteins, nucleic acids, and lipids *in vitro* and *in vivo* (30). Exosomes also have important biological functions in the immune response, inflammation, tumor growth, and infection (31). They also have less biohazardous potential, less cytotoxicity, and are not easily degraded. Therefore, exosomes have potential applications in the diagnosis, treatment, and prognosis of conditions like IBD. Previous studies have demonstrated that exosomes derived from human umbilical cord mesenchymal stem cells have profound effects in alleviating DSS-induced IBD. The exosomes may exert these effects by regulating the level of ubiquitin modification and modulating IL-7 expression in macrophages (20, 32). Extracellular vesicles secreted by hookworms can interact with host cells and attenuate the severity of 2,4,6-trinitrobenzenesulfonic acid (TNBS)-induced colitis in mice (33). Furthermore, exosomes released by interleukin-10-treated dendritic cells can inhibit TNBS-induced colitis in rats (34). The functions of exosomes in IBD primarily depend on the internal functional components and exosome-induced transport mechanisms involving cell-cell communications or cell-environment interactions. Designing new drugs by exosome-like-structure may provide a new insight into IBD treatment (35). Like exosomes, other studies also supply novel insights into potential IBD treatment, such as purified fruit bromelain, which can inhibit epithelial TNF-α receptors to ameliorate intestinal inflammation and barrier dysfunction in colitis (36). As well as that, fortunellin induced modulation of phosphatase and tensin homolog by miR-374a maintains intestinal barrier functions and decreases inflammation in TNBS-induced rat colitis (37). However, more animal and clinical research is needed to study the functions of novel insights (including exosomes) on IBD.

Macrophages are commonly classified as M1 macrophages and M2 macrophages. Previous studies have shown that macrophage exosomes affect disease processes. Exosomes released by differentially activated macrophages influence the dormancy and resurgence of breast cancer cells in the bone marrow stroma (38). In colon cancer, the M2 macrophages secrete exosomes that promote cell migration and invasion (39). Exosomes derived from tumor-associated macrophages promote the migration of gastric cancer cells via the transfer of functional apolipoprotein E (40). However, to the best of our knowledge, the role of macrophage exosomes in IBD has not yet been reported. Based on the roles of M2 macrophages in anti-inflammatory and immunoregulatory activity, we studied the combined advantages of exosomes derived from various M2 macrophage phenotypes in IBD. We found that the M2b macrophage exosomes effectively attenuated DSS-induced colitis.

The Treg cells are key regulators of immune homeostasis (41). They are important for the control of the immune response, and they alleviate defects during the development of autoimmune and chronic inflammatory conditions, such as IBD (29). The Treg cells also contribute to the complex pathogenesis of IBD during the onset and development of the disease, and patients with IBD have significantly reduced numbers of peripheral Treg cells (42, 43). Our results have shown that the percentage of Treg cells in the spleens of mice with DSS-induced colitis were increased following treatment with M2b macrophage exosomes. These results indicate that M2b macrophage exosomes had protective effects during the development of DSS-induced colitis, which were partly mediated by the up-regulation of Treg cells.

Studies have shown that levels of the pro-inflammatory cytokines, IL-1β and IL-6, are predictably increased in the mucosa of patients with Crohn's disease and ulcerative colitis (44). Mucosal immune cells are an abundant source of IL-1β (44). Furthermore, IL-6 is abundantly secreted in the intestinal mucosa of patients with IBD, and is implicated in the pathogenesis of IBD via the soluble IL-6 receptor, activation of immune cells, inhibition of apoptosis, and induction of Th17-cell differentiation (45). The expression of IL-17 is increased in the serum and mucosa of most patients with IBD, and is consistently higher in Crohn's disease than it is in ulcerative colitis (46). Our results showed that IL-1β, IL-6, and IL-17A were all down-regulated in mice with DSS-induced colitis, following treatment with M2b macrophage exosomes.

The M2b macrophages play a role in Th2 activation and immunoregulation (12, 14). The M2b macrophages are also specifically characterized by secretion of the CCL1 chemokine (47), a ligand of the cognate chemokine receptor CCR8 (48, 49). The CCR8 receptor is expressed at high levels in Treg cells and in polarized Th2 cells, both of which migrate in response to the appropriate agonist, CCL1 (50). Accordingly, CCR8 knockout mice show impaired Th2 cytokine production, and the CCL1/CCR8 axis has been implicated in the cutaneous homing of T cells (51, 52). Studies have shown that CCL1 and its receptor, CCR8, mediate the conversion of mesenchymal stem cells to embryoid bodies expressing Treg cells (53), and disruption of the CCL1-CCR8 axis inhibits vascular Treg recruitment and functions (54). In the present study, we found that M2b macrophage exosomes carrying the CCL1 protein to the colon interact with CCR8 to promote IL-4 expression (Th2 polarization) and increase Treg percentages. As an anti-inflammatory cytokine, IL-4 inhibited inflammation in IBD patients (55) and induced a Th2-type immune response in the mice with colitis. These effects, induced by IL-4, can balance the Th1-type immune response and thereby relieve colitis (56). In addition, M2b macrophage exosomes increased Treg percentages via the CCL1/CCR8 axis, which can further balance the effects of inflammatory disorders.

In summary, our findings show that M2b macrophage exosomes attenuate disease activity, up-regulate Treg cells and IL-4, and reduce pro-inflammatory cytokine (IL-1β, IL-6, and IL-17A) production in mice with DSS-induced colitis. The results also suggest that M2b macrophage exosomes exert protective effects in DSS-induced colitis, which are mainly mediated by the CCL1/CCR8 axis. Taken together, our findings provide insight into the immunobiology of M2b macrophage exosomes and suggest a useful novel approach to IBD treatment.

## Data Availability Statement

All datasets generated for this study are included in the manuscript/supplementary files.

## Ethics Statement

The animal study was reviewed and approved by The Animal Research Ethics Committee of Sun Yat-sen University.

## Author Contributions

RY, LW, XS, PH, DY, and ZW drafted this manuscript. YL and RY performed the experiments, analyses, and interpretation of the data. YH helped the aforementioned authors to develop the experiments. DY and ZW provided final approval of the version to be published. All authors discussed the complete dataset to establish an integral and coherent analysis.

### Conflict of Interest

The authors declare that the research was conducted in the absence of any commercial or financial relationships that could be construed as a potential conflict of interest.
